# RETRACTED: Cammalleri et al. A Dietary Combination of Forskolin with Homotaurine, Spearmint and B Vitamins Protects Injured Retinal Ganglion Cells in a Rodent Model of Hypertensive Glaucoma. *Nutrients* 2020, *12*, 1189

**DOI:** 10.3390/nu18132154

**Published:** 2026-07-03

**Authors:** Maurizio Cammalleri, Massimo Dal Monte, Rosario Amato, Paola Bagnoli, Dario Rusciano

**Affiliations:** 1 Department of Biology, University of Pisa, via San Zeno, 31, 56127 Pisa, Italy; maurizio.cammalleri@unipi.it (M.C.); rosario.amato@biologia.unipi.it (R.A.); paola.bagnoli@unipi.it (P.B.); 2 Interdepartmental Research Center Nutrafood “Nutraceuticals and Food for Health”, University of Pisa, via del Borghetto 80, 56124 Pisa, Italy; 3 Sooft Research Center, Viale Andrea Doria, 21, 95125 Catania, Italy; dario.rusciano@sooft.it

The journal retracts the article titled, “A Dietary Combination of Forskolin with Homotaurine, Spearmint and B Vitamins Protects Injured Retinal Ganglion Cells in a Rodent Model of Hypertensive Glaucoma” [1], cited above.

Following publication, concerns were brought to the attention of the Editorial Office regarding the integrity of a figure presented in this article [1].

In accordance with the journal’s standard procedures, an investigation was conducted by the Editorial Office and Editorial Board, that confirmed concerns in Figure 4, including inappropriate image editing and duplication between panels presented in this figure and material from two different published articles [2,3] presenting different experimental conditions. Although the authors cooperated with the investigation, the explanations and supporting materials provided were not deemed sufficient by the Editorial Board to resolve the identified concerns. As a result, the Editorial Board has lost confidence in the reliability of the findings and has decided to retract this publication [1], as per MDPI’s retraction policy.

This retraction was approved by the Editor-in-Chief of *Nutrients*.

The authors have been informed of this retraction and have indicated their agreement with this decision.
